# Synchronous bone metastasis in lung cancer: retrospective study of a single center of 15,716 patients from Tianjin, China

**DOI:** 10.1186/s12885-021-08379-2

**Published:** 2021-05-26

**Authors:** Xu Guo, Wenjuan Ma, Haixiao Wu, Yao Xu, Dezheng Wang, Shuang Zhang, Zheng Liu, Vladimir P. Chekhonin, Karl Peltzer, Jin Zhang, Xin Wang, Chao Zhang

**Affiliations:** 1 Department of Bone and Soft Tissue Tumors, Tianjin Medical University Cancer Institute and Hospital, National Clinical Research Center for Cancer, Key Laboratory of Cancer Prevention and Therapy, Tianjin’s Clinical Research Center for Cancer, Tianjin, China; 2 Department of Orthopedics, Cangzhou Central Hospital, Cangzhou, Hebei province China; 3 Department of Breast Imaging, Tianjin Medical University Cancer Institute and Hospital, National Clinical Research Center for Cancer, Key Laboratory of Cancer Prevention and Therapy, Tianjin’s Clinical Research Center for Cancer, Tianjin, China; 4 Department of Non-communicable Disease Control and Prevention, Tianjin Centers for Disease Control and Prevention, Tianjin, China; 5 Department of Orthopedics, Heilongjiang Province Hospital, Harbin, Heilongjiang Province China; 6 Department of Basic and Applied Neurobiology, Federal Medical Research Center for Psychiatry and Narcology, Moscow, Russian Federation; 7 Department of Psychology, University of the Free State, Bloemfontein, South Africa; 8 Department of Epidemiology and Biostatistics, West China School of Public Health, Sichuan University, Chengdu, Sichuan province China

**Keywords:** Lung Cancer, Synchronous bone metastasis, Prognosis, Adenocarcinoma

## Abstract

**Background:**

This study aimed to describe the incidence, clinical characteristics, and prognosis of lung cancer patients with synchronous bone metastasis (SBM) and to analyze the prognostic factors of the lung cancer patients with SBM.

**Methods:**

A total of 15,716 lung cancer patients who were diagnosed between 2009 to 2018 in the Tianjin Medical University Cancer Institute and Hospital were retrospectively reviewed. Among them, patients with SBM were checked. Both the demographic and clinical characteristics were included as follows: age, gender, marital status, history of smoking, alcohol consumption, family history of tumor, Karnofsky score, lymph node metastasis, histological type. Besides, laboratory data such as alkaline phosphatase, lactate dehydrogenase, carcinoembryonic antigen, squamous cell carcinoma antigen, cytokeratin-19 fragment, and neuron specific enolase were also included. The log-rank test and multivariate Cox regression analysis were employed to reveal the potential prognostic predictors. A further analysis using the Kaplan–Meier was employed to demonstrate the difference on the prognosis of LC patients between adenocarcinoma and non-adenocarcinoma.

**Results:**

Among the included patients, 2738 patients (17.42%) were diagnosed with SBM. A total of 938 patients (34.3%) with SBM were successfully followed and the median survival was 11.53 months (95%CI: 10.57–12.49 months), and the 1-, 2-, and 5-year overall survival rate was 51, 17, and 8%, respectively. Multivariable Cox regression results showed history of smoking and high level of NSE were associated with the poor prognosis, while adenocarcinoma histological type was associated with better survival.

**Conclusion:**

The prevalence of SBM in lung cancer is relatively high with poor survival. The lung cancer patients with SBM showed diverse prognosis. Among all the pathological types, the division of adenocarcinoma suggested different prognosis of the lung cancer patients with SBM. The present study emphasized the importance of pathological diagnosis on prognostic determinants in lung cancer patients with SBM.

## Background

Lung cancer (LC) has become the most common cancer and the leading cause of cancer-related deaths in the world. Aapproximate 13% of the estimated new cancers and 24% of estimated deaths were caused by LC in 2019 [1].

Due to a special microenvironment in the bone matrix, bone was accepted to be one of the most common distal metastatic sites, especially for LC [2]. A retrospective cohort study reported a total of 245 patients (19.1%) suffered bone metastasis (BM) among 1283 LC patients [3]. A higher incidence (28.2%) in LC was also reported by Oliveira MB et al. [4] and 36.9% in small cell lung cancer by Conen K [5]. LC patients with BM were usually with frustrating quality of life, resulted by the occurrence of skeletal-related events (SREs), including severe pain, orthopedic surgery interventions, palliative radiation to the bone, hypercalcaemia, pathologic bone fractures, and spinal cord compression. A total of 62.6% of non-small cell lung cancer (NSCLC) patients showed at least one SREs and 16.8% of them showed multiple SREs [6]. The optimal treatment of SREs was accepted to be the early treatment and prevention of BM. Thus, the study looking into BM in LC is warrant.

BM in lung cancer can be found at diagnosis, while minority can be found in their later course after diagnosis [7]. Synchronous bone metastasis (SBM) and metachronous bone metastasis (MBM) were previously defined as different types of BM. Few studies looking into the differences between SBM and MBM in LC were performed. However, SBM and MBM in LC may represent distinct clinicopathological characteristics, therapeutic sensitivity, and prognostic outcomes [8]. Such difference resulted in the individualized treatment plans.

Compared with the LC patients with MBM, a significant tumor burden and a complicated organism destroy in patients with SBM can usually be found [9]. Thus, the patients with SBM usually suffer more mental stress and financial burden. The accurate prognostic determinants are of significance on generalizing individualized clinical decision.

A series of prognosis prediction models for BM were reported and employed. Ignoring the difference between SBM and MBM, the revised Tokuhashi score system for spinal metastasis classified lung cancer as score 0, indicating the worst prognosis. Such classification neglected the effect of histological type on the survival of LC patients [10, 11]. Lately, Tokuhashi suggested that the system should include serum biomarkers, which can improve the predictive ability and the accuracy of survival estimation [12]. In the revised Katagiri system, lung cancer patients with BM were divided into two groups according to the treatment with molecularly targeted drugs. Those LC patients with molecularly targeted drugs were classified as moderate growth tumor, while those without targeted drugs were classified as rapid growth tumor [13]. However, seldom patients with SBM were diagnosed with molecularly targeted drugs. Our previous study, based on Surveillance, Epidemiology, and End Results (SEER) database, reported different survivals in various histological types of LC patients with BM [14]. The results suggested the histological type was one of the independent prognostic factors for LC patients with SBM. Considering the racial difference between the east and west, we performed the present research to further study SBM in LC.

In the present study, we conducted a comprehensive analysis on the survival and clinical characteristics in a large cohort of LC patients with SBM. We also investigated the factors that being associated with SBM occurrence and prognosis, which could help the clinicians predict the prognosis and tailor targeted treatment regimens for lung cancer patients with SBM.

## Methods

**Fig. 1:**
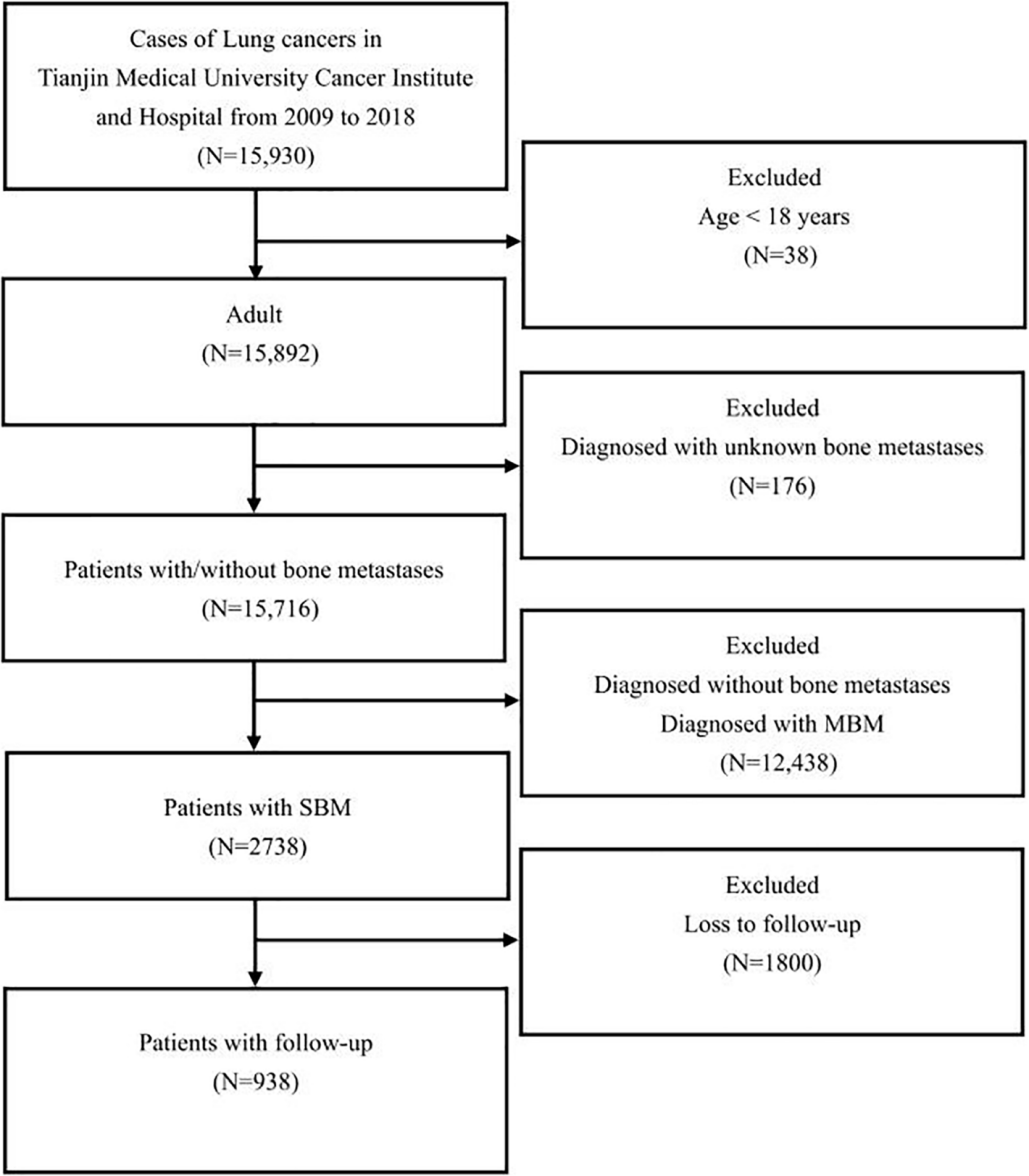
The flow-chart of the selection for lung cancer patients with synchronous bone metastasis

This retrospective analysis was approved by the Ethics Committee of Tianjin Medical University Cancer Institute & Hospital. The medical records of LC patients were electronically and manually checked. Between January 2009 and December 2018, a total of 15,930 LC patients were initially diagnosed in our hospital. The patients younger than 18 years old or with uncertain bone metastasis were excluded, 15,716 LC patients were retrieved. Among them, LC patients with SBM were chosen for prognostic analysis. The exclusion criteria were (1) those who were diagnosed without BM; (2) those who were diagnosed with MBM in LC; (3) those who were not followed during follow-up. Patients were followed through clinic and telephone. Death was further confirmed by linking the death register system of Tianjin Centers for Disease Control. SBM was defined as BM diagnosis within 3 months with LC diagnosis, while MBM was defined as BM diagnosis more than 3 months after LC diagnosis. The flow-chart of the subjects’ selection was shown in Fig. 1.

### Statistical analysis

Patients’ demographic and clinical characteristics were included as follows: age (18–45 years, 46–65 years or > 65 years), gender (female or male), marital status (married and other status or unmarried), history of smoking (yes or none), alcohol consumption (yes or none), family history of cancer (yes or none), Karnofsky score (10–40, 50–70, or 80–100), lymph node metastasis (yes or none), histological type (small cell lung cancer, adenocarcinoma, squamous cell carcinoma, large cell lung cancer, mixed lung cancer or others). Laboratory data for SBM patients were also investigated. The median level of the data was defined as threshold value, including alkaline phosphatase (ALP: < 102.00 mmol/L or ≥ 102.00 mmol/L), lactate dehydrogenase (LDH: < 215.00 U/L or ≥ 215.00 U/L), carcinoembryonic antigen (CEA: < 15.64 ng/ml or ≥ 15.64 ng/ml), squamous cell carcinoma antigen (SCC: < 0.80 ng/ml or ≥ 0.80 ng/ml), cytokeratin-19 fragment (Cyfra21–1: < 4.93 ng/ml or ≥ 4.93 ng/ml), neuron specific enolase (NSE: < 16.67 ng/ml or ≥ 16.67 ng/ml).

The overall survival was analyzed using the Kaplan–Meier method and the difference was tested by the log-rank test. Multivariable Cox regression model, including significant univariate factors (*P < 0.05*) was conducted for analyzing the independent prognostic factors for LC patients with SBM. According to the results, a further analysis using the Kaplan–Meier method was employed to demonstrate the LC patients’ prognosis differences between adenocarcinoma and non-adenocarcinoma. All statistical analyses were performed using SPSS 23.0 (IBM Corporation, Armonk, NY) and all charts on survival were conducted by MedCalc 15.2.2. Two-sided *P < 0.05* were considered as statistically significant.

## Results

### Demographic data

Among the included patients, 2738 (17.42%) were diagnosed with SBM. A total of 938 LC patients with SBM (550 males and 388 females) were with reliable prognosis of follow-up. The mean age of these patients was 61.41 ± 9.94 years. Except the patients without explicit histological type (40.40%), the most common histological type is adenocarcinoma in 331 patients (35.29%), followed by squamous cell carcinoma (13.21%), small-cell lung cancer (SCLC) (7.36%), mixed carcinoma (2.35%), and large-cell carcinoma in 13 patients (1.39%). All detailed information of the included patients was summarized in Table 1.

**Table 1:** Demographic information of the included patients

Clinical subjects	Number of patients	Proportion (%)
**Age, (years)**		
18–45	51	5.44
46–65	572	60.98
> 65	315	33.58
**Gender**		
Female	388	41.36
Male	550	58.64
**Marital status**		
Married	933	99.47
Unmarried	5	0.53
**History of smoking**		
None	436	46.48
Yes	501	53.41
Unknown	1	0.11
**Alcohol consumption**		
None	703	74.95
Yes	234	24.94
Unknown	1	0.11
**Family history of tumor**		
None	754	80.38
Yes	183	19.51
Unknown	1	0.11
**KPS**		
10–40	7	0.75
50–70	128	13.65
80–100	395	42.11
Unknown	408	43.49
**Lymph node metastasis**		
None	368	39.23
Yes	537	57.25
Unknown	33	3.52
**Pathology**		
Small-cell	69	7.36
Adenocarcinoma	331	35.29
Squamous cell	124	13.22
Large cell	13	1.38
Mixed	22	2.34
Others (unknown)	379	40.41
**ALP**		
< 102.00 mmol/L	347	36.99
≥ 102.00 mmol/L	355	37.85
Unknown	236	25.16
**LDH**		
< 215.00 U/L	420	44.78
≥ 215.00 U/L	438	46.70
Unknown	80	8.52
**CEA**		
< 15.64 ng/ml	404	43.07
≥ 15.64 ng/ml	405	43.18
Unknown	129	13.75
**SCC**		
< 0.80 ng/ml	402	42.86
≥ 0.80 ng/ml	404	43.07
Unknown	132	14.07
**Cyfra21–1**		
< 4.93 ng/ml	401	42.75
≥ 4.93 ng/ml	401	42.75
Unknown	136	14.50
**NSE**		
< 16.67 ng/ml	401	42.75
≥ 16.67 ng/ml	402	42.86
Unknown	135	14.39

*KPS* Karnofsky score, *ALP* Alkaline phosphatase, *LDH* Lactate dehydrogenase, *CEA* Carcinoembryonic antigen, *SCC* Squamous cell carcinoma antigen, *Cyfra21–1* Cytokeratin-19 fragment, *NSE* Neuron specific enolase

### Survival rates and prognostic factors

**Fig. 2:**
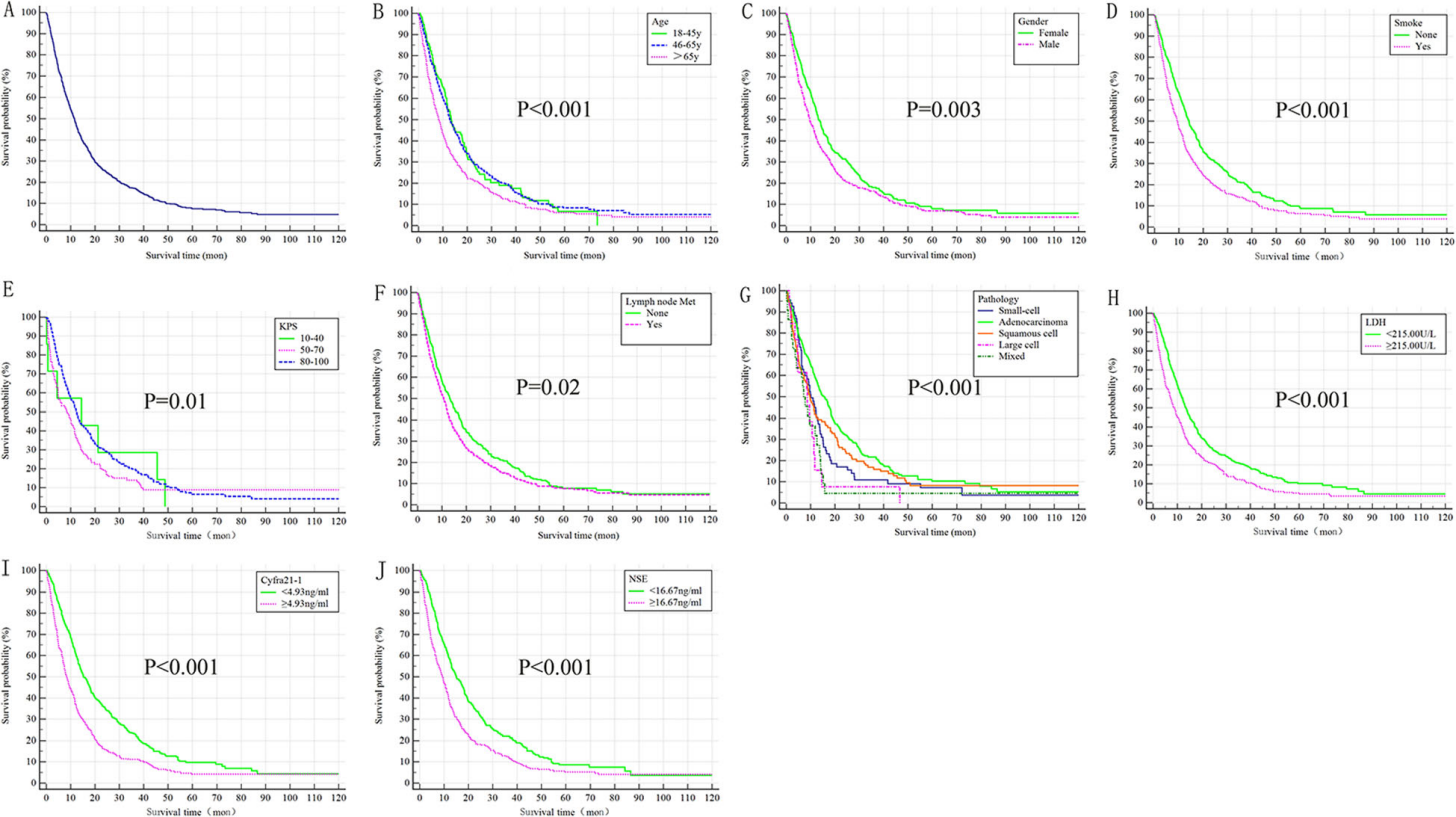
The survival curves for lung cancer patients with synchronous bone metastasis (**A**, overall), stratified by age (**B**), gender (**C**), smoke (**D**), KPS (**E**), lymph node metastasis (**F**), pathology (**G**), LDH (**H**), Cyfra21–1 (**I**) and NSE (**J**)

The median survival time of 938 patients was 11.53 months (95%CI: 10.57–12.49 months). The 1-, 2-, and 5-year overall survival rate was 51, 17, and 8%, respectively. Survival curves for patients was shown in Fig. 2A.

Log-rank test showed the overall survival in subjects with older age (Fig. 2B, *P < 0.001*), male (Fig. 2C, *P = 0.003*), history of smoking (Fig. 2D, *P < 0.001*), KPS = 50–70 (Fig. 2E, *P = 0.010*), with lymph node metastasis (Fig. 2F, *P = 0.020*), higher level of LDH (Fig. 2H, *P < 0.001*), Cyfra21–1 (Fig. 2I, *P < 0.001*) and NSE (Fig. 2J, *P < 0.001*) were worse than that with the counterparts (Table 2). The patients of adenocarcinoma presented significantly better overall median survival (15.67 months, 95%CI: 13.22–18.12) (Fig. 2G, *P < 0.001*).

**Table 2:** The significant prognostic factors after log-rank test

Clinical subjects	Median survival (95%CI), mons	HR (95%CI)	***P-value***
**Age, (years)**			*0.20*
18–45	11.90 (10.08–13.72)	1 (Reference)	*1.00*
46–65	12.97 (11.65–14.28)	0.85 (0.45–1.60)	*0.61*
> 65	8.13 (6.70–9.57)	1.12 (0.58–2.17)	*0.81*
**Gender**			
Female	13.43 (11.95–14.92)	1 (Reference)	*1.00*
Male	9.63(8.44–10.83)	1.04 (0.75–1.47)	*0.80*
**History of smoking**			
None	14.13 (12.42–15.85)	1 (Reference)	*1.00*
Yes	9.23 (7.92–10.55)	1.42(1.02–1.96)	*0.04*
Unknown	NA	NA	NA
**KPS**			*0.67*
10–40	14.47 (0.00–40.30)	1 (Reference)	*1.00*
50–70	8.30 (4.88–11.72)	2.28 (0.77–6.79)	*0.14*
80–100	12.43 (11.12–13.74)	1.94 (0.67–5.63)	*0.22*
Unknown	NA	NA	NA
**Lymph node metastasis**			
None	13.37 (11.33–15.40)	1 (Reference)	*1.00*
Yes	10.77 (9.56–11.98)	1.10 (0.81–1.45)	*0.51*
Unknown	NA	NA	NA
**Pathology**			*0.03*
Small-cell	10.10 (7.48–12.73)	1.22 (0.78–1.91)	*0.38*
Adenocarcinoma	15.67 (13.22–18.12)	1 (Reference)	*1.00*
Squamous cell	9.73 (7.62–11.85)	1.42 (0.98–2.05)	*0.06*
Large cell	8.67 (2.99–14.34)	1.85 (0.80–4.30)	*0.15*
Mixed	7.10 (4.15–10.05)	2.09 (1.11–3.94)	*0.02*
Unknown	NA	NA	*NA*
**LDH**			
< 215.00 U/L	13.43 (11.90–14.96)	1 (Reference)	*1.00*
≥ 215.00 U/L	8.67 (7.37–9.97)	1.12 (0.82–1.51)	*0.48*
Unknown	NA	NA	*NA*
**Cyfra21–1**			
< 4.93 ng/ml	15.33 (13.36–17.31)	1 (Reference)	*1.00*
≥ 4.93 ng/ml	8.30 (7.17–9.43)	1.26 (0.93–1.71)	*0.13*
Unknown	NA	NA	*NA*
**NSE**			
< 16.67 ng/ml	15.00 (12.74–17.27)	1 (Reference)	*1.00*
≥ 16.67 ng/ml	9.23 (7.86–10.61)	1.42 (1.06–1.92)	*0.02*
Unknown	NA	NA	*NA*

*KPS* Karnofsky score, *LDH* Lactate dehydrogenase, *Cyfra21–1* Cytokeratin-19 fragment, *NSE* Neuron specific enolase

**Fig. 3:**
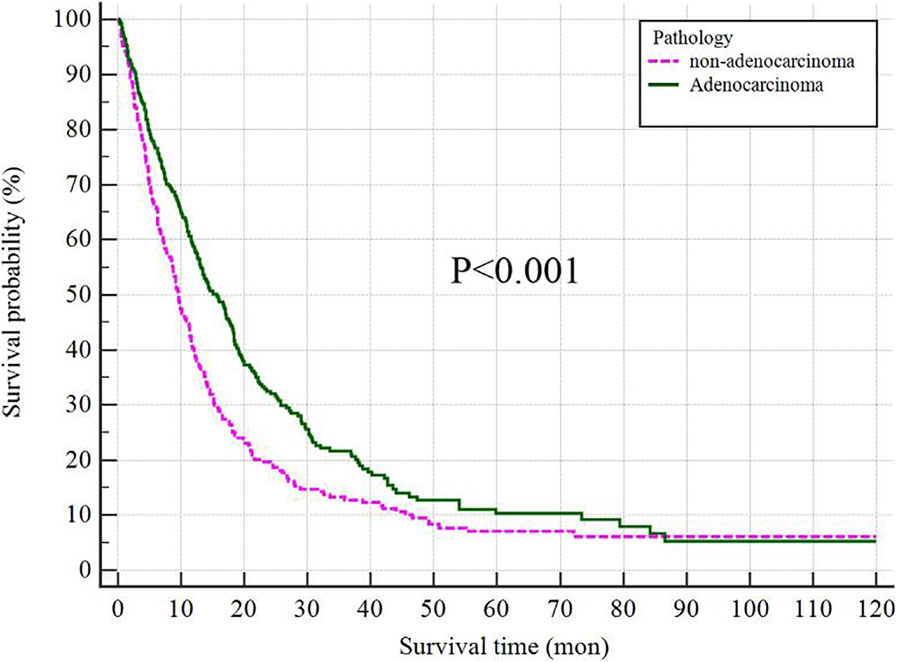
The survival curves for lung cancer patients with adenocarcinoma and non-adenocarcinoma

Multivariable Cox regression results suggested the patients with history of smoking and high level of NSE were associated with poor prognosis. LC Patients with adenocarcinoma were associated with better survival in patients with SBM. The overall median survival of LC adenocarcinoma patients with SBM was 15.67 months (95%CI: 13.22–18.12), while that in non-adenocarcinoma was 9.73 months, (95%CI: 7.62–11.85) (Fig. 3, *P < 0.001*).

## Discussion

To our knowledge, based on the largest single center population, the present research studied SBM in patients with LC. A total of 2738 LC patients (17.42%) were diagnosed with SBM in 15,716 LC patients. Such incidence was less than our previous reported incidence (20.9%) in a study based on the data from the SEER dataset [14].

The present study suggested the median survival of LC patients with SBM was 11.53 months. Different levels of survival between the patients with synchronous and metachronous bone metastasis were reported [7, 15–17]. It was reported the survival of 18.04 months in LC patients with SBM and 9.12 months in LC patients with MBM [8]. The criteria studying synchronous and metachronous metastasis in cancer was still indefinite. For the cancer patients with metachronous metastasis, the survival can be defined from the initial diagnosis of primary cancer to death, while from the diagnosis of metastasis to death [9, 18, 19]. This may be the main cause of the different results of OS between synchronous and metachronous metastasis in previous studies [8, 9]. The potential explanation for the better survival in cancer patients with synchronous metastasis than those with metachronous metastasis was the occurrence of chemotherapy resistance in the patients with metachronous metastasis [20]. Such resistance significantly limited the treatment choice of the first-line chemotherapy [8]. To get the data standardized, the present study defined OS being within 3 months from BM diagnosis, instead of lung cancer diagnosis. Such standardized definition can make sense to the prediction of prognosis and clinical treatment decisions based on the clinical features in LC patients with SBM.

Three prognostic factors in LC patients with SBM were found, including smoking, high level of NSE and histological type. Recently, Tokuhashi et al. suggested that the predictive model should include serum biomarkers [12]. Some serum factors were reported to be correlated with the prognosis, including the levels of ALP, and LDH [21, 22]. However, in our study with the large population, we did not find the significant correlation between the prognosis and most blood test factors. A series of commonly used indicators were involved, including ALP, LDH, CEA, SCC, Cyfra21–1 and NSE. NSE was proved to be the only indicator with significant effect on the survival of LC patients with SBM. NSE was previously proved to be an important indicator for tumor aggressiveness and bone metastasis development [23, 24]. NSE was found to be one of the prognostic factors in metastatic prostate cancer [25]. Thus, NSE can be a potential prognostic predictor in LC patients with BM.

Another independent prognostic factor of LC patient with SBM was smoking. It was concluded that smoking affected various organs and was a leading cause of premature disease and death [26]. The metabolism of tobacco carcinogens, variations in nicotine receptor-related genes, inflammatory response to the tobacco-induced lung damage and DNA repair were reported to be the consequences of the smoking [27–30]. Smoking was reported to potentially inhibit chemo- and radiotherapy response [31]. Thus, smoking was widely accepted to be a prognostic factor of LC patients [32]. Our study further proved it was one of the prognostic factors of LC patients with SBM.

The median survival of adenocarcinoma LC with SBM was 15.67 months, while that of non-adenocarcinoma LC was 9.63 months *(P < 0.001*). Compared with other histological types in LC patients, poor survival of LC patients with non-adenocarcinoma was reported. The frustrating survival might be caused by poor response from the tyrosine kinase inhibitors (TKIs) therapy [33]. In previous studies, to properly manage the patients with cancer, the cancer patients were separated into the slow (estimated survival> 20 months), moderate (estimated survival 10 to 20 months), and rapid (estimated survival< 10 months) growth groups [13, 34, 35]. Therefore, LC adenocarcinoma patients with SBM should be treated as the guideline for moderate growth group, while non-adenocarcinoma with SBM as rapid growth group. Thus, the clarification pathologic diagnosis of LC is of significant importance for the prognostic determinants in LC patients with SBM.

In our previous study using the SEER data, the median survival of LC with SBM was 4.00 months. Histological type and number of the metastatic sites (brain, lung and liver metastasis) were proved to be the independent prognosis factors [14]. When stratified by different histological types, the OS of adenocarcinoma was significantly longer than OS of other histological types, which was consistent with the present study. Survival difference between our previous SEER study and the present study was potentially resulted by the cohort with different regional and ethnic. Another potential explanation for such difference may result in the developed treatment for LC with SBM in recent years. Compared with the present study, SEER public database did not provide the information on performance status, smoking status, and serum biomarkers such as ALP, LDH, CEA, SCC, Cyfra21–1 and NSE. In both studies with SEER database and single center database, histological type was proved to be one of the independent prognostic factors in LC with SBM. However, Tokuhashi and Tomita scores roughly treated lung cancer as rapid growth tumor. Based on the present study, lung cancer should be categorized into different classifications, adenocarcinoma as moderate growth group, and non-adenocarcinoma as rapid growth group.

Several limitations of this study should be mentioned: (1) detailed information on the numbers and locations of bone metastasis were not recorded; (2) the present study was a single-center retrospective study, thus the bias in the program might be exist; (3) external validation was needed to further verify the results.

## Conclusions

This study evaluated the incidence of SBM in LC, reported the clinical features and prognosis of LC patients with SBM, and identified a series of prognostic factors in LC patients with SBM. The survival of LC patients with SBM was of significant difference. To properly predict the prognosis, we suggested the importance of clarification pathologic diagnosis of lung cancer in LC patients with SBM. The division of adenocarcinoma patients in LC patients with SBM can significantly guide the management of disease and aid clinicians in properly allocating medical resources to the patients.

## Data Availability

The datasets generated and/or analysed during the current study are not publicly available but are available from the corresponding author on reasonable request.

## Abbreviations

SBM: Synchronous Bone Metastasis
LC: Lung Cancer
BM: Bone Metastasis
SREs: Skeletal-Related Events
NSCLC: Non-Small Cell Lung Cancer
MBM: Metachronous Bone Metastasis
SEER: Surveillance, Epidemiology, And End Results

## References

[1] Siegel RL, Miller KD, Jemal A (2019). Cancer statistics, 2019. CA Cancer J Clin.

[2] Roato I (2014). Bone metastases: when and how lung cancer interacts with bone. World J Clin Oncol.

[3] Silva GT, Silva LM, Bergmann A, Thuler LC (2019). Bone metastases and skeletal-related events: incidence and prognosis according to histological subtype of lung cancer. Future Oncol.

[4] Oliveira MB, Mello FC, Paschoal ME (2016). The relationship between lung cancer histology and the clinicopathological characteristics of bone metastases. Lung Cancer.

[5] Conen K, Hagmann R, Hess V, Zippelius A, Rothschild SI (2016). Incidence and predictors of bone metastases (BM) and skeletal-related events (SREs) in small cell lung Cancer (SCLC): a Swiss patient cohort. J Cancer.

[6] Sun JM, Ahn JS, Lee S, Kim JA, Lee J, Park YH, Park HC, Ahn MJ, Ahn YC, Park K (2011). Predictors of skeletal-related events in non-small cell lung cancer patients with bone metastases. Lung Cancer.

[7] Pruksakorn D, Phanphaisarn A, Settakorn J, Arpornchayanon U, Tantraworasin A, Chaiyawat P, klangjorhor J, Teeyakasem P (2018). Prognostic score for life expectancy evaluation of lung cancer patients after bone metastasis. J Bone Oncol.

[8] Sohn S, Chung CK, Jung JH, Lee KC, Kim J, Chang UK, Sohn MJ, Kim SH (2018). Nationwide comparative study of synchronous and metachronous spine metastasis in the adult Korean population. J Clin Neurosci.

[9] Fleckenstein J, Petroff A, Schafers HJ, Wehler T, Schope J, Rube C (2016). Long-term outcomes in radically treated synchronous vs metachronous oligometastatic non-small-cell lung cancer. BMC Cancer.

[10] Tokuhashi Y, Matsuzaki H, Toriyama S, Kawano H, Ohsaka S (1990). Scoring system for the preoperative evaluation of metastatic spine tumor prognosis. Spine (Phila Pa 1976).

[11] Tokuhashi Y, Matsuzaki H, Oda H, Oshima M, Ryu J (2005). A revised scoring system for preoperative evaluation of metastatic spine tumor prognosis. Spine (Phila Pa 1976).

[12] Tokuhashi Y, Uei H, Oshima M, Ajiro Y (2014). Scoring system for prediction of metastatic spine tumor prognosis. World J Orthop.

[13] Katagiri H, Okada R, Takagi T, Takahashi M, Murata H, Harada H, Nishimura T, Asakura H, Ogawa H (2014). New prognostic factors and scoring system for patients with skeletal metastasis. Cancer Med.

[14] Zhang C, Mao M, Guo X, Cui P, Zhang L, Xu Y, Li L, Han X, Peltzer K, Xiong S, Baklaushev VP, Wang X, Wang G (2019). Nomogram based on homogeneous and heterogeneous associated factors for predicting bone metastases in patients with different histological types of lung cancer. BMC Cancer.

[15] He YF, Luo HQ, Wang W, Chen J, Yao YW, Cai SB (2015). Clinical features and prognosis-associated factors of non-small cell lung cancer exhibiting symptoms of bone metastasis at the time of diagnosis. Oncol Lett.

[16] Bae HM, Lee SH, Kim TM, Kim DW, Yang SC, Wu HG, Kim YW, Heo DS (2012). Prognostic factors for non-small cell lung cancer with bone metastasis at the time of diagnosis. Lung Cancer.

[17] Cetin K, Christiansen CF, Jacobsen JB, Norgaard M, Sorensen HT (2014). Bone metastasis, skeletal-related events, and mortality in lung cancer patients: a Danish population-based cohort study. Lung Cancer.

[18] Ma CX, Guan X, Wei R, Wang S, Quan JC, Zhao ZX (2020). The distinction of clinicopathological characteristics, treatment strategy and outcome in colorectal cancer patients with synchronous vs metachronous bone metastasis. Front Oncol.

[19] Slesser AA, Georgiou P, Brown G, Mudan S, Goldin R, Tekkis P (2013). The tumour biology of synchronous and metachronous colorectal liver metastases: a systematic review. Clin Exp Metastasis.

[20] Drizou M, Kotteas EA, Syrigos N (2017). Treating patients with ALK-rearranged non-small-cell lung cancer: mechanisms of resistance and strategies to overcome it. Clin Transl Oncol.

[21] Lang J, Zhao Q, He Y, Yu X (2018). Bone turnover markers and novel biomarkers in lung cancer bone metastases. Biomarkers..

[22] Sunaga T, Shimamoto K, Nakamura S, Takahashi N, Higashino M, Hozumi T, Matsui M, Nagatani A, Kokubu F, Kogo M, Sasaki T (2017). The association between fever and prognosis in lung cancer patients with bone metastases receiving Zoledronic acid. Chemotherapy..

[23] Maeda T, Ueoka H, Tabata M, Kiura K, Shibayama T, Gemba K, Takigawa N, Hiraki A, Katayama H, Harada M (2000). Prognostic factors in advanced non-small cell lung cancer: elevated serum levels of neuron specific enolase indicate poor prognosis. Jpn J Clin Oncol.

[24] Zhou Y, Chen WZ, Peng AF, Tong WL, Liu JM, Liu ZL (2017). Neuron-specific enolase, histopathological types, and age as risk factors for bone metastases in lung cancer. Tumour Biol.

[25] Kamiya N, Akakura K, Suzuki H, Isshiki S, Komiya A, Ueda T, Ito H (2003). Pretreatment serum level of neuron specific enolase (NSE) as a prognostic factor in metastatic prostate cancer patients treated with endocrine therapy. Eur Urol.

[26] Murray CJ, Atkinson C, Bhalla K, Birbeck G, Burstein R, Chou D, Dellavalle R, Danaei G, Ezzati M, Fahimi A, Flaxman D, Foreman, Gabriel S, Gakidou E, Kassebaum N, Khatibzadeh S, Lim S, Lipshultz SE, London S, Lopez, MacIntyre M, Mokdad AH, Moran A, Moran AE, Mozaffarian D, Murphy T, Naghavi M, Pope C, Roberts T, Salomon J, Schwebel DC, Shahraz S, Sleet DA, Murray, Abraham J, Ali MK, Atkinson C, Bartels DH, Bhalla K, Birbeck G, Burstein R, Chen H, Criqui MH, Dahodwala, Jarlais, Ding EL, Dorsey ER, Ebel BE, Ezzati M, Fahami, Flaxman S, Flaxman AD, Gonzalez-Medina D, Grant B, Hagan H, Hoffman H, Kassebaum N, Khatibzadeh S, Leasher JL, Lin J, Lipshultz SE, Lozano R, Lu Y, Mallinger L, McDermott M, Micha R, Miller TR, Mokdad AA, Mokdad AH, Mozaffarian D, Naghavi M, Narayan KM, Omer SB, Pelizzari PM, Phillips D, Ranganathan D, Rivara FP, Roberts T, Sampson U, Sanman E, Sapkota A, Schwebel DC, Sharaz S, Shivakoti R, Singh GM, Singh D, Tavakkoli M, Towbin JA, Wilkinson JD, Zabetian A, Murray, Abraham J, Ali MK, Alvardo M, Atkinson C, Baddour LM, Benjamin EJ, Bhalla K, Birbeck G, Bolliger I, Burstein R, Carnahan E, Chou D, Chugh SS, Cohen A, Colson KE, Cooper LT, Couser W, Criqui MH, Dabhadkar KC, Dellavalle RP, Jarlais, Dicker D, Dorsey ER, Duber H, Ebel BE, Engell RE, Ezzati M, Felson DT, Finucane MM, Flaxman S, Flaxman AD, Fleming T, Foreman, Forouzanfar MH, Freedman G, Freeman MK, Gakidou E, Gillum RF, Gonzalez-Medina D, Gosselin R, Gutierrez HR, Hagan H, Havmoeller R, Hoffman H, Jacobsen KH, James SL, Jasrasaria R, Jayarman S, Johns N, Kassebaum N, Khatibzadeh S, Lan Q, Leasher JL, Lim S, Lipshultz SE, London S, Lopez, Lozano R, Lu Y, Mallinger L, Meltzer M, Mensah GA, Michaud C, Miller TR, Mock C, Moffitt TE, Mokdad AA, Mokdad AH, Moran A, Naghavi M, Narayan KM, Nelson RG, Olives C, Omer SB, Ortblad K, Ostro B, Pelizzari PM, Phillips D, Raju M, Razavi H, Ritz B, Roberts T, Sacco RL, Salomon J, Sampson U, Schwebel DC, Shahraz S, Shibuya K, Silberberg D, Singh JA, Steenland K, Taylor JA, Thurston GD, Vavilala MS, Vos T, Wagner GR, Weinstock MA, Weisskopf MG, Wulf S, Murray, U.S. Burden of Disease Collaborators (2013). The state of US health, 1990-2010: burden of diseases, injuries, and risk factors. JAMA..

[27] Hu Z, Wang Y, Wang X, Liang G, Miao X, Xu Y, Tan W, Wei Q, Lin D, Shen H (2005). DNA repair gene XPC genotypes/haplotypes and risk of lung cancer in a Chinese population. Int J Cancer.

[28] Fathy M, Hamed M, Youssif O, Fawzy N, Ashour W (2014). Association between environmental tobacco smoke exposure and lung cancer susceptibility: modification by antioxidant enzyme genetic polymorphisms. Mol Diagn Ther.

[29] Yu Y, Liu H, Zheng S, Ding Z, Chen Z, Jin W, Wang L, Wang Z, Fei Y, Zhang S, Ying K, Zhang R (2014). Gender susceptibility for cigarette smoking-attributable lung cancer: a systematic review and meta-analysis. Lung Cancer.

[30] Cho YJ, Cho YM, Kim SH, Shin KH, Jung ST, Kim HS (2019). Clinical analysis of patients with skeletal metastasis of lung cancer. BMC Cancer.

[31] Czyzykowski R, Polowinczak-Przybylek J, Potemski P (2016). Nicotine-induced resistance of non-small cell lung cancer to treatment--possible mechanisms. Postepy Hig Med Dosw (Online).

[32] Liu X, Jiang T, Li W, Li X, Zhao C, Shi J, Zhao S, Jia Y, Qiao M, Zhang L, Luo J, Gao G, Zhou F, Wu F, Chen X, He Y, Ren S, Su C, Zhou C (2018). Characterization of never-smoking and its association with clinical outcomes in Chinese patients with small-cell lung cancer. Lung Cancer.

[33] Wang D, Luo Y, Shen D, Yang L, Liu HY, Che YQ (2019). Clinical features and treatment of patients with lung adenocarcinoma with bone marrow metastasis. Tumori..

[34] Tomita K, Kawahara N, Kobayashi T, Yoshida A, Murakami H, Akamaru T (2001). Surgical strategy for spinal metastases. Spine (Phila Pa 1976).

[35] Katagiri H, Takahashi M, Wakai K, Sugiura H, Kataoka T, Nakanishi K (2005). Prognostic factors and a scoring system for patients with skeletal metastasis. J Bone Joint Surg Br.

